# Editorial: Protein Glycosylation—Advances in Identification, Characterization and Biological Function Elucidation Using Mass Spectrometry

**DOI:** 10.3389/fchem.2022.847242

**Published:** 2022-02-18

**Authors:** Ganglong Yang, Hui Zhang, Wen Yi, Shi Yan, Liwei Cao

**Affiliations:** ^1^ The Key Laboratory of Carbohydrate Chemistry & Biotechnology, Ministry of Education, School of Biotechnology, Jiangnan University, Wuxi, China; ^2^ Department of Pathology, Johns Hopkins University, Baltimore, MD, United States; ^3^ Department of Chemical and Biomolecular Engineering, Johns Hopkins University, Baltimore, MD, United States; ^4^ Department of Hepatobiliary and Pancreatic Surgery, Zhejiang Provincial Key Laboratory of Pancreatic Disease, The First Affiliated Hospital, School of Medicine, Zhejiang University, Hangzhou, China; ^5^ Institut für Parasitologie, Veterinärmedizinische Universität, Wien, Austria

**Keywords:** protein glycosylation, mass spectrometry, glycoproteomics, glycomics, SARS-CoV-2

Glycosylation is one of the most common post-translational modifications, with over 50% of human proteins having been reported to be glycosylated. The identification and functional validation of protein glycosylation are important to reveal the roles of glycoproteins in biological processes. Until now, fewer than 20% of predicted glycoproteins in Uniprot were experimentally verified. Therefore, a comprehensive, rapid and sensitive methodology to map the protein glycosylation events is required more than ever.

A number of high-quality articles relevant to characterizing glycoproteins are reported in our Research Topic “Protein Glycosylation-Advances in Identification, Characterization and Biological Function Elucidation using Mass Spectrometry,” which describe a variety of experimental techniques especially high-resolution mass spectrometry (MS). At first, fast and highly specific enrichment methods for glycoprotein/glycopeptides preparation are imperative for broad application of glycoprotein analysis in high-throughput clinical samples. Fan et al. developed an ultrafast sample preparation method for N-glycoproteome using thermoresponsive magnetic fluid-immobilized enzymes, in which protein digestion and deglycosylation of glycopeptide were done within 3 min. Chen et al. developed a method to sequentially enrich glycopeptides and phosphopeptides using a TiO_2_ material, which were then sequentially subjected to MS for characterization of glycosylation sites and phosphosites, respectively. The two methods facilitated comprehensive analyses of N-linked glycosylation events by MS.

Glycosylation is critical for physiological and pathological functions of glycoproteins. Tumor growth benefits from the ability of cancer cells to bypass cellular division checkpoints, evade death signals and immune surveillance, and migrate to metastatic sites. Glycosylation plays critical roles in all of these processes (Reily et al., 2019). In order to dissect the functional roles of glycosylation in human diseases, new technologies are needed for improvement of the depth and breadth of glycoproteome analysis, especially for the characterization of intact glycopeptides. Analysis of intact glycopeptides could provide information regarding to glycosylation sites, glycan compositions at specific glycosylation sites of glycoproteins. It preserves biological context of the modification and enables us to understand proteome-wide glycan heterogeneity. In this Research Topic, Tabang et al. comprehensively reviewed the advances in MS-based technologies focusing on characterization of glycome and glycoproteome of diabetes, pancreatitis, and pancreatic cancer. The future of MS-based methods was forecasted to discover new protein targets for early detection of human diseases. Liu et al. developed a N-linked and O-linked glycosylation mapping method to identify and quantify the occurrence of each glycoform of human plasma fibronectin in a site-specific manner, in which CID and stepped normalized collision energy (sNCE)-HCD tandem MS were deployed. These site-specific glycosylation patterns of fibronectin in human plasma can facilitate functional analyses and development of corresponding therapeutics. Zhao et al. integrated the proteomic and glycoproteomic data to identify potential clinical (glyco)protein targets for early detection of type 2 diabetes (T2D). They identified alterations of site-specific N-linked glycosylation related to the complement activation pathways in T2D patients, which were not observed by corresponding proteome measurements. It demonstrated that MS-based glycoproteomics method is a powerful tool to understand (pre)diabetes. Shu et al. applied the lectin blot and intact O-linked glycopeptide MS analysis to discover aberrant O-glycosylation of Haptoglobin in hepatocellular carcinoma, revealing up-regulation of most O-glycopeptides in HCC patient serum. The study by Demus et al. sheds new insights on glycosylation of apo-CIII that is able to regulate the triglyceride clearance, facilitating our understanding of the role of apo-CIII in the regulation of lipid metabolism in various disease settings.

Glycomics has become more essential and significant in the cancer studies and glycans were deemed as potential biomarkers in cancer. The liquid biopsies, e.g., blood and urine, are great sources for screening and characterization of targets for early detection of human diseases. Moreover, mass spectrometry imaging (MSI) is a well-established technique to spatially map biomolecules across fresh frozen or formalin-fixed paraffin-embedded (FFPE) tissue sections (Briggs et al., 2019). Blaschke et al. analyzed the N-glycome of urine, urine EPS, prostatic fluids, urine EPS-derived extracellular vesicles using MALDI-MS, and the N-glycan profile of prostatic tissue using MSI. Over 100 N-linked glycan compositions were detected, and a subset of N-glycans present in fluids were found to be derived from the gland lumens. The developed N-glycan profiling method is able to analyze large clinical cohorts, and is adaptable to characterize other biofluids. Shu et al. deployed the lectin microarray and MS to analyze protein glycosylation of saliva of the patients with Esophageal squamous cell carcinoma (ESCC). The specific glycopatterns, e.g., sialylation and fucosylation, were significantly altered in ESCC patients in comparison to healthy controls, and DSA detection was thought to be a potential diagnostic tool.

SARS-CoV-2, the causative pathogen of COVID-19, induces fever, severe respiratory illness, and pneumonia (Watanabe et al., 2020), which caused approximate 270 million infections and more than 5 million deaths until now. The SARS-CoV-2 S gene encodes a glycoprotein with 22 potential N-linked glycosites and dozens of potential O-linked glycosites, which likely play critical roles in protein folding and immune evasion. Wang et al. compared the N-glycosylation profiles of recombinant S proteins produced by CHO and HEK cells, revealing higher levels of complex type and sialylation type of glycans on CHO-expressed S protein and a decreased level of high-mannose glycans on this protein relative to the S protein produced in HEK cells. Zhang et al. compared the O-glycosylation profiles of recombinant S proteins produced by insect and HEK cells using HCD and EThcd MS-based methods, and found that most of the O-glycosites in S protein from human cells were sialylated. To comprehensively analyze O-glycosylation of S protein, Cui et al. developed an O-glycopeptide enrichment method with a dual-functional histidine-bonded silica material, resulting in identification of 46 O-glycosites.

Due to the microheterogeneity and nonlinear structural complexity of glycans, characterization of glycan structures is one of challenge tasks in the field. MS is a powerful tool to analyze the glycan structures and there is a great progress partially facilitated by the advance of instruments in the past decades. However, processing and characterization of numerous fragmental glycan spectrum from MS raw files are still challenging. Zhang et al. developed an automatically GUI-based tool (GlycanGUI) for annotation and quantification of glycan compositions. GlycanGUI could interpretate the data generated by different separation columns, MS instruments and/or buffers, and even different laboratories. Huang et al. presented an automated tool for processing MALDI-MS-based glycan isotope labeling data (gQuant), which was designed with a set of dedicated algorithms, including spectral preprocessing, glycan mapping, quantitation, and ratio calculation. Wang et al. also presented a new algorithm (hepPareser) to decipher the main components of Low-molecular-weight heparins based on the LC/MS data. These tools will be greatly beneficial for structural and functional studies of protein glycosylation.

O-GlcNAcylation is a prevalent form of posttranslational modifications on the hydroxyl group of serine and/or threonine residues (Torres and Hart, 1984). The systematical characterization of O-GlcNAcylation is needed for revealing its functional roles in human diseases. Zhu and Yi reviewed chemistry-assisted methods for characterization of O-GlcNacylation, which provided comprehensive insights for the labeling and identification of O-GlcNAcylation. Yin et al. systematically reviewed the MS-based methodologies for qualitative or quantitative characterization of O-GlcNAcylation.

Glycans from glycoconjugates are synthesized through a series of reactions mediated by glycosyltransferases (GTs) or glycoside hydrolases (GHs). The CRISPR-Cas9 technology enables us to edit the glyco-related gene expression, and thus facilitates our understanding of the functions of glycosylation in cells. Yang et al. applied the intact glycopeptides analysis method to reveal differential expression of core-fucosylation between normal CHO cells and CHO cells with Fut8 knock-out, revealing knock-out of FUT8 influenced core-fucosylation of glycoproteins as well as other processes of glycosylation synthesis, resulting in alteration of protein glycosylation. With this, the relationship of glycan compositions, structures and modified proteins could be mapped (Narimatsu et al., 2019; Huang et al., 2021).

In conclusion, the articles collected in this Research Topic will facilitate the identification, characterization and functional elucidation of protein glycosylation, and pave the way to future ambitious challenges in glycobiology.
